# Effects on the Physical and Mechanical Properties of Porous Concrete for Plant Growth of Blast Furnace Slag, Natural Jute Fiber, and Styrene Butadiene Latex Using a Dry Mixing Manufacturing Process

**DOI:** 10.3390/ma9020084

**Published:** 2016-01-29

**Authors:** Hwang-Hee Kim, Chun-Soo Kim, Ji-Hong Jeon, Chan-Gi Park

**Affiliations:** 1 Research institute of technology, Nature and Environment Co. Ltd., 116-28 Boheung 1-Gil, Kongju 325-33, Korea; hwanghekim@hanmail.net (H.-H.K.); nkimcs@chol.com (C.-S.K.); 2 Department of Environmental Engineering, Andong National University, 1375 Gyeongdong Street, Andong 760-749, Korea; jhjeon@anu.ac.kr; 3 Department of Rural Construction Engineering, Koungju National University, 54 Daehak Street, Yesan 340-702, Korea

**Keywords:** by product materials, blast furnace slag aggregates, blast furnace slag powder, mechanical properties, latex polymer, void ratio, porous concrete for plant growth

## Abstract

To evaluate the effects of industrial by-products materials on the performance of porous concrete for plant growth, this study investigated the physical, strength, and freeze/thaw resistances of porous concrete for plant growth, prepared by replacing cement with blast furnace slag powder at 60% by weight, and replacing natural stone aggregates with coarse blast furnace slag aggregates at rates of 0%, 20%, 40%, 60% and 100% by weight. In addition, the effects of adding natural jute fiber and styrene butadiene **(**SB) latex to these concrete mixtures were evaluated. The void ratio, compressive strength, and freeze/thaw resistance of the samples were measured. With increasing replacement rate of blast furnace aggregates, addition of latex, and mixing of natural jute fiber the void ratio of the concrete was increased. Compressive strength decreased as the replacement rate of blast-furnace slag aggregates increased. The compressive strength decreased after 100 freeze/thaw cycles, regardless of the replacement rate of blast furnace slag aggregates or of the addition of natural jute fiber and latex. The addition of natural jute fiber and latex decreased the compressive strength after 100 freeze/thaw cycles. The test results indicate that the control mixture satisfied the target compressive strength of 10 MPa and the target void ratio of 25% at replacement rates of 0% and 20% for blast furnace aggregates, and that the mixtures containing latex satisfied the criteria up to an aggregate replacement rate of 60%. However, the mixtures containing natural jute fiber did not satisfy these criteria. The relationship between void ratio and residual compressive strength after 100 freeze/thaw cycles indicates that the control mixture and the mixtures containing jute fiber at aggregate replacement rates of 20% and 40% satisfied the target void ratio of 25% and the target residual compressive strength of over 80% after 100 freeze/thaw cycles. The mixtures containing latex and aggregate replacement rates up to 60% satisfied the target void ratio and compressive strength.

## 1. Introduction

As interest in ecological restoration has increased in Korea and other countries, diverse research has been conducted into appropriate concrete design. In particular, studies on porous concrete for plant growth have demonstrated a number of practical achievements in terms of research results and ecological restoration data [1,2,3,4]. A 40%–60% by weight replacement of cement with a fine powder of blast furnace slag, an industrial by-product, is effective in reducing both the use of cement and the alkalinity of concrete [5,6]. In addition, the long-term addition of fine blast furnace slag powder promotes plant growth by aiding the neutralization of concrete. The replacement of cement with fine slag can also contribute to mitigation of global warming [7,8]. Worldwide, CO_2_ emissions generated by cement production account for about 3% of total global carbon emissions [8,9], and production of one ton of cement generates about 870 kg of CO_2_. Hence, a reduction in the use of cement will decrease CO_2_ emission, which is a major cause of global warming [7,8]. Rock-crushed stone has been widely used as an aggregate in concrete production; however, the use of crushed stone has environmental and conservation impacts, and there is an increasing need for research into other aggregates [9]. Blast furnace slag aggregates may be one such replacement. As with fine powder slag, blast furnace slag aggregates are waste by-products of the steel industry [10]. Thus, replacing rock-crushed stone with blast furnace slag aggregates is considered to be of use both in environmental protection and resource recycling. Thus far, blast furnace slag aggregates have been used extensively as substrates or as auxiliary base floor materials; a great deal of research has been conducted in recent years into their use as concrete aggregates. In particular, recent results indicate significant potential for using blast furnace slag aggregates in concrete due to their material properties [11]. Porous concrete for plant growth is manufactured using a dry process, which enables form removal immediately after fabrication of the concrete specimen and may demonstrate increased productivity compared with wet processes that require removal of concrete forms after a period of time has elapsed. In the dry process, however, because the form is removed immediately, problems such as changing shape or dripping of concrete may occur if the concrete has some fluidity. Additionally, if the amount of mixing water is too small, the hydration reaction may not proceed sufficiently and problems such as difficult rod tamping may occur during specimen fabrication. It is thus important to determine an appropriate amount of mixing water. In this study, blast furnace slag powder was used to replace up to 60% of the cement weight in concrete fabrication, and the effects of adding styrene butadiene (SB) latex and natural jute fiber were evaluated. Additionally, the effect of the replacement rate of crushed stone aggregates with coarse aggregates of blast furnace slag on the physical and strength performance of porous concrete was investigated.

## 2. Materials

### 2.1. Materials

**Figure 1:**
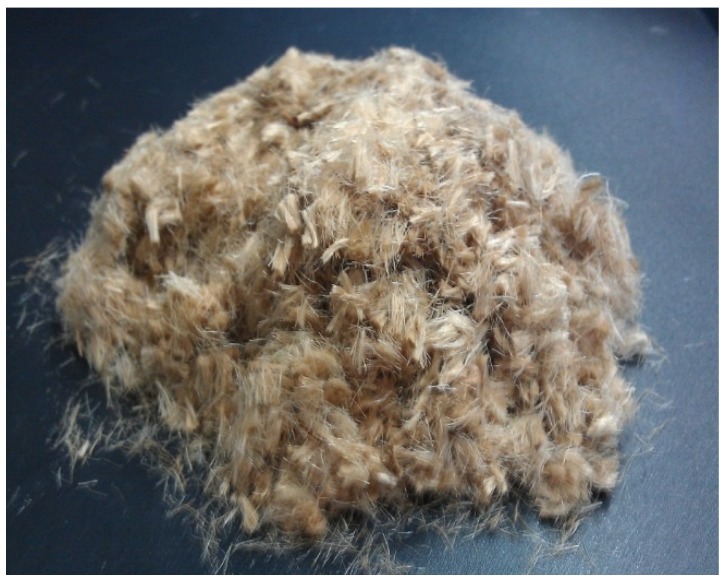
Photo of jute fiber.

ASTM Type 1 cement, fine blast furnace slag powders, and coarse blast furnace slag aggregates were used in this study. The properties of each material are given in Table 1, Table 2 and Table 3, respectively. In general, concrete prepared using blast furnace slag aggregates has a similar compressive strength to concrete based on natural crushed stone or gravels, but a higher absorbance [10]. Thus, it is necessary to conduct initial mixing while maintaining surface dry saturated conditions for the blast furnace slag aggregates. Natural jute fiber was also used, which contains surface hydroxyl radicals and has excellent adhesion to concrete. The shape and properties of the fiber are shown in Figure 1 and Table 4, respectively. SB latex was also used in this study. SB latex enhances the adhesion between materials due to the formation of a latex film, thus increasing the strength properties of porous concrete. The properties of latex are given in Table 5.

**Table 1:** Properties of cement.

Fineness (cm^2^/g)	Specific Gravity	Stability (%)	Setting Time		Compressive Strength (MPa)		
			Initial (min)	Final (min)	3 days	7 days	28 days
3200	3.15	0.02	220	400	20	30	38

**Table 2:** Chemical composition of blast furnace slag.

Chemical Composition (%)							
SiO_2_	Al_2_O_3_	Fe_2_O_3_	CaO	MgO	MnO	TiO	S
33.1	13.9	0.29	42.4	6.1	0.4	0.96	0.66

**Table 3:** Physical properties of coarse aggregate.

Type of Aggregate	Maximum Size (mm)	Specific Gravity	Absorption (%)	Fineness Modulus
Natural aggregate	25	2.65	0.35	6.92
Blast furnace slag aggregate	25	2.36	5.0	6.80

**Table 4:** Properties of natural jute fiber.

Elastic Modulus (GPa)	Specific Gravity	Fiber Length (mm)	Fiber Diameter (mm)	Tensile Strength (MPa)	Surface
61	1.26	3	0.015	510	Hydrophilic

**Table 5:** Properties of latex.

Solids Content (%)	Styrene Content (%)	Butadiene Content (%)	pH	Specific Gravity	Surface Tension (dyne/cm)	Particle Size (A)	Viscosity (cps)
46.5	34 ± 1.5	66 ± 1.5	11.0	1.02	30.57	1700	42

### 2.2. Mix Proportions

The effects of varying mixtures of porous concrete for plants growth produced using a dry process were investigated: natural coarse aggregates were replaced with coarse blast furnace slag aggregates, and fine blast furnace slag powder was used as a replacement for cement. The physical and mechanical properties upon addition of natural jute fiber and latex were also evaluated. Table 6 shows the mixing ratios of each component in samples used in this study. The use of cement was minimized by replacing 60% of cement by weight with fine blast furnace slag powder for all mixtures. In addition, 0.1% by volume of natural jute fiber and 5% by volume of latex were added to some sample mixtures, and natural coarse aggregates were replaced by blast furnace slag aggregates over a range of replacement rates.

In the dry process, low unit water in the mixing of materials is very difficult. The latex was added in order to ensure liquidity. In addition, the latex was added in order to improve the mixing of the material. In addition, the latex increases the attractive force to form a latex film between aggregate and aggregate.

**Table 6:** Mix proportions of porous concrete for plant growth. SB = Styrene butadiene.

Type of Mix	Unit Weight (kg/m^3^)						
	Water	Cement	Blast Furnace Slag	Natural Coarse Aggregate	Blast Furnace Slag Aggregate	Natural Jute Fiber	SB Latex
No. 1	84	128	192	1200	-	-	-
No. 2	84	128	192	960	240	0	-
No. 3	84	128	192	720	480	0	-
No. 4	84	128	192	480	720	0	-
No. 5	84	128	192	240	960	0	-
No. 6	84	128	192	-	1200	0	-
No. 7	84	128	192	1200	-	1.2	-
No. 8	84	128	192	960	240	1.2	-
No. 9	84	128	192	720	480	1.2	-
No. 10	84	128	192	480	720	1.2	-
No. 11	84	128	192	240	960	1.2	-
No. 12	84	128	192	-	1200	1.2	-
No. 13	72	128	192	1200	-	-	16
No. 14	72	128	192	960	240	-	16
No. 15	72	128	192	720	480	-	16
No. 16	72	128	192	480	720	-	16
No. 17	72	128	192	240	960	-	16
No. 18	72	128	192	-	1200	-	16

The target performance criteria for porous concrete for plant growth are a void ratio ≥25%, a compressive strength ≥10 MPa, and a residual compressive strength of 80% after 100 freeze-thaw cycles. Performance goals of the study were determined by the performance standard of the Korea Ministry of Environment for Environmental Mark Certification [12]. To fabricate experimental specimens, cement and blast furnace slag aggregates and powder were added to a mixer. Dry mixing (cement + blast furnace slag + coarse aggregate) was conducted for 30 s. After dry mixing, water was added and further mixing was conducted for 90 s. Finally, natural jute fiber and latex were added, and the specimens were mixed for an additional 1 min. The total mixing time was 3 min. The dry process is formulated with no slump. Therefore, minimal water is added to the blended material. This study added water at 84 kg/m^3^. Additionally, latex was added. Addition of latex can increase the dispersion of the fiber by increasing the fluidity.

**Figure 2:**
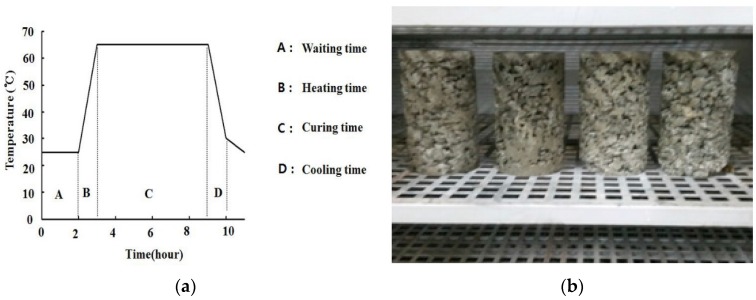
Curing of porous concrete for plant growth. (**a**) Accelerating curing process; (**b**) Curing chamber.

After mixing, the porous concrete for plant growth was poured into a mold for fabrication of experimental specimens, and rod tamping was conducted. Rod tamping was undertaken using a compactor exerting pressure. After rod tamping, the form of the specimens was removed, and accelerated curing was performed using a thermo-hygrostat. Currently in Korea, an accelerated curing of the porous concrete for plant growth has been conducted in production methods. This study was performed to accelerate curing. Figure 2 shows the curing process of porous concrete for plant growth—2 h exposure to air at 25 °C, and after that the temperature was raised to 65 °C for 1 h. Additionally it was then maintained at 65 °C for 6 h. Then the temperature was reduced to 25 °C for 1 h. After exposure to the thermo-hygrostat for 12 h, the sample was removed. Curing chamber conditions the total exposure time is one day.

## 3. Test methods

### 3.1. Void Ratio

Void ratio was measured in cylindrical specimens (diameter: 100 mm; height: 200 mm) using the volumetric method from the Eco-Blocks Study Committee of the Japanese Blocks Industry Association. The percentage void ratio was calculated after 28 days after accelerated curing (10 h), curing in air (26 days), and curing in water (1 day) , using the following equation:

(1) $$P_{a}=1-\frac{W_{2}-W_{1}}{V}\times 100$$

where *W*_1_ and *W*_2_ are the underwater and air-dried weights of the specimen, respectively, and *V* is the volume of the specimen.

The specimens were prepared such that the pores were saturated with water as much as possible. After water curing for one day before the test, the specimens were measured based on their dry weight after measuring the weight of the water.

### 3.2. Compressive Strength

Compressive strength tests were carried out according to the ASTM C39 standard [13], to evaluate the effects of the jute fiber fraction, latex and blast furnace slag aggregate replacement rate. Cylindrical specimens (diameter: 100 mm; height: 200 mm) were fabricated and underwent accelerated curing (10 h) and curing in air (27 days) prior to the compressive strength test.

### 3.3. Repeated Freezing and Thawing Cycles Test

To evaluate the freeze/thaw resistance of the porous concrete for plant growth, a freezing-thawing repetition test of the material after accelerated curing (10 h) and curing in air (27 days) was conducted according to the ASTM C666 standard [14]. The freeze/thaw experiment was conducted by cooling the sample from 4 to –18 °C then raising the temperature to 4 °C, for 100 cycles. A compressive strength test was then conducted to measure the residual compressive strength.

## 4. Results

### 4.1. Void Ratio

**Figure 3:**
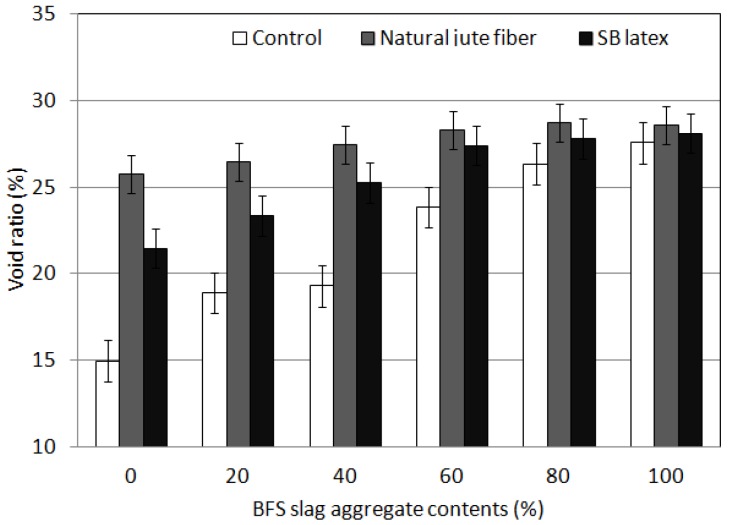
Void ratio results of porous concrete for plant growth.

The void ratio test results shown in Figure 3 indicate that the void ratio increased as the replacement rate of blast-furnace slag aggregates increased; this is because blast furnace slag aggregates are porous materials. The addition of natural jute fiber also increased the void ratio. However, the "fiber ball" phenomenon, arising from the presence of multiple fibers, was observed, because the fluidity of the mixtures was sufficiently small so that it was impossible to accomplish sufficient dispersion of the fibers. The void ratio test results after addition of latex indicate that latex caused a relative increase in void ratio. The target void ratio (more than 25%) [12] was satisfied for 80% and 100% of coarse blast furnace slag aggregate content in the “Control” mix without the addition of natural jute fiber or latex. The target void ratio of 25% (more than 25%) was achieved for all mixtures that included natural jute fiber for coarse stone and blast furnace slag aggregates. For mixtures containing latex, the target void ratio (more than 25%) was achieved for mixtures with a replacement rate of over 40% (the contents of blast-furnace slag aggregates).

### 4.2. Compressive Strength

Blast furnace slag aggregate porosity is larger compared with that of general aggregate. This property offers an advantage when applying the porous concrete for plant growth. It is also environmentally friendly via its use of industrial by-products. Therefore, this study applied a blast furnace slag aggregate.

**Figure 4:**
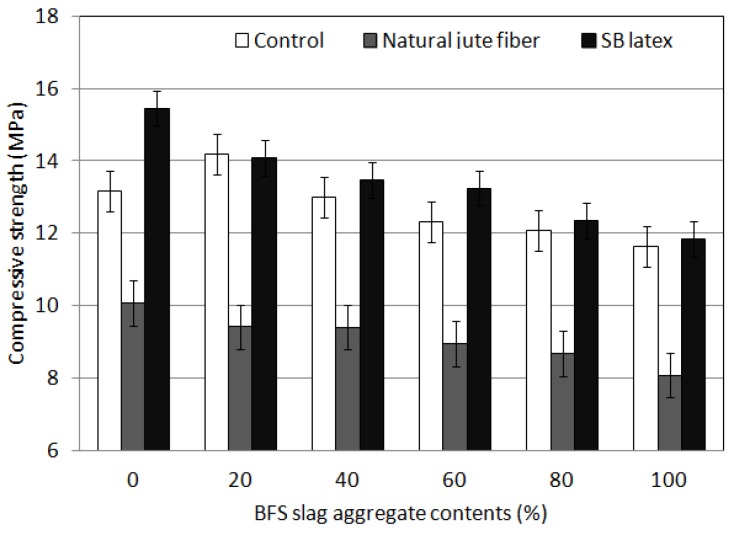
Compressive strength results of porous concrete for plant growth.

The results of the compressive strength test are shown in Figure 4. The test results indicate that the compressive strength of the porous concrete for plant growth decreased as the replacement rate of blast-furnace slag aggregates (the contents of blast-furnace slag aggregates) increased. At a replacement rate of 20% (content of blast-furnace slag aggregates), however, the compressive strength increased. These results indicate that because the void ratio of blast-furnace slag aggregates is greater than that of natural coarse aggregates, the compressive strength decreased due to an increase in the number of pores. At 20% replacement (content of blast-furnace slag aggregates), however, the increase in void ratio was still small; thus, the long-term strength increased. Because the porous concrete for plant growth was cured through initial steam curing and then 28 days of water curing, a long-term curing effect was created resulting in a long-term strength increase for the blast furnace slag aggregates. At a replacement rate of 40% (content of blast-furnace slag aggregates), however, the strength decreased through an increase in void ratio. The addition of natural jute fiber decreased the strength. These results indicate that because the dry mixing process could not achieve sufficient fluidity to suppress the fiber ball phenomenon, a decrease in strength was observed. Thus, some method of increasing the initial fluidity must be used when fiber reinforcements are included in the concrete mix. The addition of latex increased the compressive strength of the porous concrete for plant growth blocks. Latex enables the smooth coating of binders around aggregates by increasing the initial fluidity and increasing the adhesion between aggregates through adhesive forces, and hence improves the compressive strength. In addition, the target design strength of over 10 MPa [12] was satisfied for all mixtures excluding those with natural jute fiber added; the target strength of 10 MPa was not achieved in mixtures with fiber added for all blast furnace slag aggregate replacement rates.

### 4.3. Repeated Freezing and Thawing Cycles

**Figure 5:**
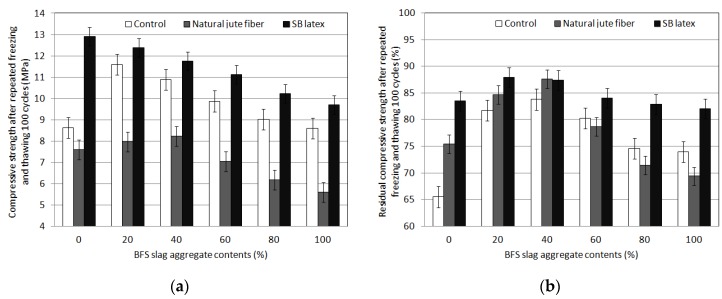
Repeated freezing and thawing cycles test results of porous concrete for plant growth. (**a**) Compressive strength; (**b**) Residual compressive strength.

The results of the freeze/thaw resistance test are shown in Figure 5. The compressive strength after a material age of 28 days and 100 freeze/thaw cycles decreased, regardless of the replacement rate of blast-furnace slag aggregates or the addition of natural jute fiber or latex. In general, in the case of a porous concrete with high void ratio, the compressive strength decreases significantly. If natural jute fiber is added, however, the generation and growth of cracks at the interface between the coarse aggregates caused by freeze/thaw repetitions is suppressed. Because natural jute fiber is a hydrophilic material with excellent adhesion to binders due to strong hydrogen bonding, adhesion at interfaces is increased and the decrease in compressive strength after freeze/thaw cycles is small. In this study, however, the dry process could not achieve sufficient freeze/thaw resistance due to the fiber ball phenomenon. The freeze/thaw resistance results after the addition of latex indicate that latex alleviates the decrease in compressive strength after freeze/thaw cycles, due to reinforcement through coating of binders in the interface between coarse aggregates, due to the increase in initial fluidity and increased adhesion between materials. The results of the residual compressive strength test after freeze/thaw repetitions indicate that the target residual compressive strength of over 80% after 100 freeze/thaw cycles was achieved in all mixtures with latex added. In addition, mixtures with natural jute fiber added and the control mixture satisfied the target residual compressive strength of 80% at replacement rates of 20% and 40% (content of blast-furnace slag aggregates).

### 4.4. Relationship between Compressive Strength and Void Ratio

**Figure 6:**
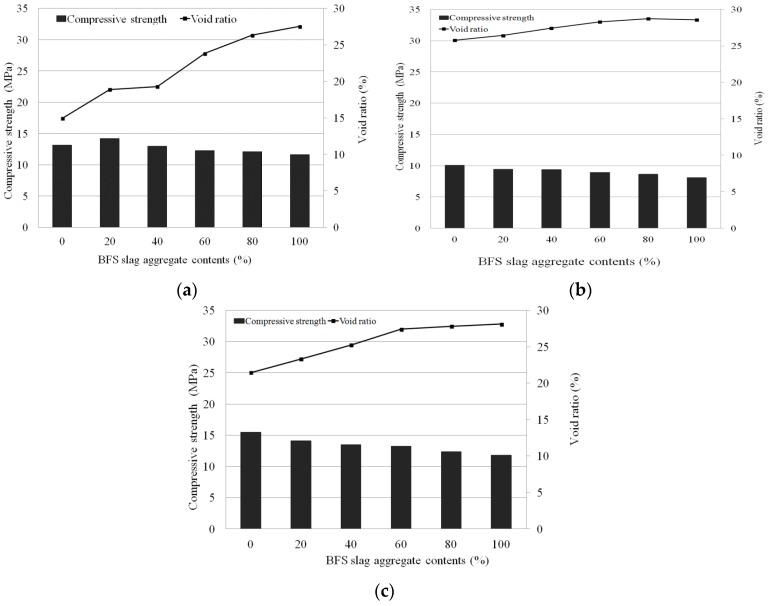
Relationship compressive strength and void ratio of porous concrete for plant growth. (**a**) Control; (**b**) Natural jute fiber; (**c**) SB latex.

The relationship between compressive strength and void ratio is shown in Figure 6. As the compressive strength decreased, the void ratio increased. In general, when the void ratio increases, the compressive strength decreases [15,16,17,18] regardless of the replacement rate of blast-furnace slag aggregates or the presence of added natural jute fiber or latex.

Porous concrete for plant growth contains pores to activate plant growth. Thus, its compressive strength will be significantly smaller than that of non-porous concrete. In general, therefore, if promotion of vegetation is the main goal, the void ratio rather than the strength should be improved for ecological restoration. However, for concrete for plant growth applied to rivers and lakes, it is necessary to improve both the void ratio and the strength, as safety and plant growth should be satisfied simultaneously. Here, the effect of blast-furnace slag aggregates and natural jute fiber and latex on the compressive strength of porous concrete for plant growth has been investigated. Fiber reinforcements may increase the number of binders in the interface between aggregates and suppress the generation and growth of cracks in the interface through their cross-linking effect, enhancing compressive strength. In addition, latex can improve destruction resistance and strength by increasing the adhesive force and suppressing poor tamping and poor coating of binders on the surface of aggregates.

While the control mixture satisfied the target compressive strength of 10 MPa and the target void ratio of 25% at replacement rates of 80% and 100%, other mixtures could not meet these criteria. In the mixtures containing natural jute fiber, however, as the replacement rate of blast-furnace slag aggregates increased, the decrease in compressive strength and increase in void ratio was large, although the compressive strength was below the target strength of 10 MPa. The mixtures containing latex met the target compressive strength and void ratio above a replacement rate of 40% (more than) for blast-furnace slag aggregates.

### 4.5. Relationship between Cyclic Freeze/Thaw Resistance and Void Ratio

**Figure 7:**
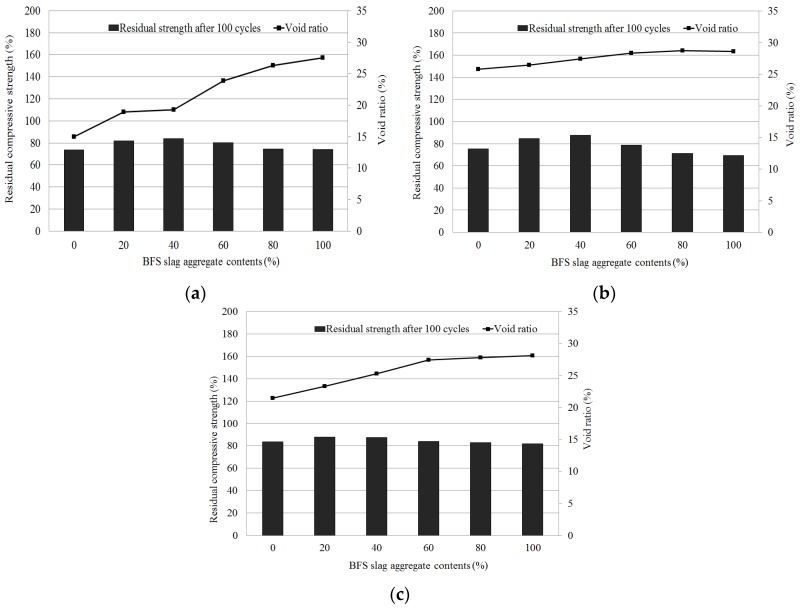
Relationship repeated freezing and thawing cycles test results and void ratio of porous concrete for plant growth. (**a**) Control; (**b**) Natural jute fiber; (**c**) SB latex.

The relationship between void ratio and compressive strength after 100 freeze/thaw cycles is shown in Figure 7. As the replacement rate of blast-furnace slag aggregates increased, the void ratio increased and the compressive strength after 100 freeze/thaw cycles also decreased. In addition, the mixtures containing natural jute fiber at replacement rates of 20% and 40% met the void ratio and compressive strength targets after 100 freeze/thaw cycles. In the mixtures containing latex, the void ratio and compressive strength targets were satisfied at replacement rates above 60% (more than 60%) for blast-furnace slag aggregates.

## 5. Conclusions

In this study, the effects of replacing natural stone aggregates with blast-furnace slag aggregates and addition of natural jute fiber and latex on porous concrete for plant growth were investigated, by adding fine blast furnace slag powders and coarse blast-furnace slag aggregates.

As the replacement rate of blast-furnace slag aggregates increased, the void ratio increased. This phenomenon occurs because the void ratio of blast-furnace slag aggregates is greater than that of natural aggregates. The addition of natural jute fiber also increased the void ratio. The void ratio test results after the addition of latex indicate that the addition of latex increased the void ratio. In the mixtures which did not contain natural jute fiber or latex, the target void ratio of over 25% was achieved at replacement rates of 80% and 100% for blast-furnace slag aggregates. All of the mixtures containing natural jute fiber met the design void ratio.

The compressive strength of the porous concrete for plant growth decreased as the replacement rate of blast-furnace slag aggregates increased. At a replacement rate of 20%, however, the compressive strength increased. The addition of latex increased the compressive strength of the porous concrete for plant growth. The compressive strength test results indicate that the targeted design strength of over 10 MPa was achieved by all mixtures, except for those containing natural jute fiber.

The compressive strength after 100 freeze/thaw cycles decreased regardless of the replacement rate of blast-furnace slag aggregates, and whether or not natural jute fiber or latex were added. The addition of natural jute fiber, a reinforcement, decreased the compressive strength after 100 freeze/thaw cycles. The addition of latex decreased the compressive strength after the freeze/thaw cycles.

The results of the compressive strength and void ratio tests indicate that the control mixture achieved the target compressive strength of 10 MPa and target void ratio of 25% at replacement rates of 80% and 100% for blast-furnace slag aggregates. In the mixtures containing latex, these criteria were satisfied at a replacement rate above 40% (more than) for blast-furnace slag aggregates.

The relationship between void ratio and residual compressive strength indicates that the mixtures which satisfied the target void ratio of 25% and the target residual compressive strength of over 80% after 100 freeze/thaw cycles were the control mixtures, the mixtures containing natural jute fiber—replacement rates of 20% and 40% for blast-furnace slag aggregates—and the mixtures containing latex at replacement rates more than 40%.
